# Supplementary material for: “Live a normal life”: Constructions of resilience among people in mixed HIV status relationships in Canada
Source: PLoS One. 2023 Mar 8;18(3):e0281301. doi: 10.1371/journal.pone.0281301 (PMC9994671; doi:10.1371/journal.pone.0281301)
Supplement: S1 Appendix — Qualitative interview guides. (PDF) [file pone.0281301.s001.pdf]

Appendix: Positive Plus One Study interview guides

This document contains the four interview guides used in the Positive Plus One study in-depth qualitative interviews. The interviews were developed and tested to probe similar topics for all four goups, and the probes and sub-topics were often contextually different for each.

1. HIV-negative partner in a current mixed HIV-status relationship,
2. HIV-positive partner in a current mixed HIV-status relationship,
3. HIV-negative partner in a past mixed HIV-status relationship, and
4. HIV-positive partner in a past mixed HIV-status relationship.

Qualitative Interview Topic Guide – Current Relationships
HIV-Negative Partner

Introduction (After obtaining informed consent)<br>We are interested in learning about your thoughts and experiences while involved in your current relationship; and how your relationship has changed over the course of time. Remember, there are no right or wrong answers, we are interested in your personal thoughts and experiences. And thanks in advance for your time and participation.		
A	History and characterization of serodiscordant relationships<br>First we would like to know a bit about your current relationship, for example how and when your relationship started, whether you also have any other partners, and when and how HIV entered your relationship.	
	Questions	Probes
	1. Can you tell me a little about your current relationship?	- How do they describe their current relationship<br>- Are they living together/married; if not, how often do they meet, where do they meet, and what do they do

		- What is the nature and history of their current relationship – how would they describe their partner; how/when did they meet; are they sexually and/or emotionally intimate; are they in a heterosexual, same sex, bisexual or transgendered relationship<br>-
	2. Are you seeing anyone else? Can you tell me about that relationship?	- Are they involved in any concurrent relationship(s)<br>- What is the nature and history of their other relationship(s) – how would they describe their other partner(s); how/when did they meet; are they sexually and/or emotionally intimate; are they in a heterosexual, same sex, bisexual or transgendered relationship<br>- Do they think their partner is involved in any concurrent relationships, how do they feel about that
	3. Can you tell me about your previous relationships?	- For HIV-negative partner, focus on nature of prior relationships in general, and any previous serodiscordant relationships<br>- How/why did the relationship(s) end<br>- How are things different in the current relationship
	4. Can you tell me about when you first faced HIV in the relationship?	- When in the relationship (or before) was the HIV-positive partner diagnosed<br>- When and how did the HIV-negative partner become aware of it<br>- How did that go Did it change over time<br>-
B	Satisfaction, challenges to wellbeing (individually and as a couple) and mechanisms of resiliency<br>Couples can experience many different challenges or issues in their relationships, including HIV, what types of challenges have you experienced in your relationship, and have you been able to overcome them?	
	1. Can you tell me about any issues or challenges that you have experienced in your current relationship?	- What were some of their early experiences in the relationship<br>- What about more recent relationship challenges/issues<br>- Focus on both HIV and non-HIV related issues<br>- What challenges does serodiscordance bring over and above that of gender, sexual orientation and HIV for a person not in a relationship?<br>- Ask for examples – what was the most recent issue that came up, what was the last argument about<br>- Suggest examples if none forthcoming (e.g., general relationship trials/conflicts, gender/sexual orientation issues, experience during HIV diagnosis/disclosure, treatment initiation/adherence/side effects, early/current sexual practices and

		protective strategies, concerns/fears about transmission, decisions about children/conception)<br>- What are their thoughts/feelings about these issues/challenges<br>- How/do they affect their mood/day-to-day life or sexual/relationship intimacy<br>- How do they think their partner perceives these issues/challenges
	2. How have you dealt with these issues?	- When/why did particular issues emerge, and how were they dealt with<br>- What issues were successfully resolved and how<br>- How were they managed or dealt with by the other partner<br>- Did they draw upon support from outside the relationship (e.g., friends, family, professional services)?<br>- Did similar issues/challenges arise in earlier serodiscordant relationships? If so how did they differ this time? Did they lead to break-ups in previous relationships?
	3. How do you think your current relationship is going?	- On what do they base their assessment? How can they tell when things are going well or not? What do they expect, need, want to be content/happy?<br>- Has HIV changed their conception of what they are looking for in a relationship?<br>- How has that satisfaction changed over time, how did it change with challenges related to HIV and non-HIV-related challenges<br>- How do they think their partner views their relationship now, and over the same issues and challenges<br>- If going well, why do they think it is going so well? If not going well, why do they think that is?
	4. Tell me a little about your/partner's current health. How do you think this has changed since your partner's diagnosis?	- How is their physical health and general mood<br>- What specific issues/concerns do they have in relation to HIV and otherwise<br>- For HIV-negative partner, How does the positive partner's health impact on the negative partner<br>- For HIV-negative partner, How often and when/why do they go to their partner's medical appointments with them<br>-
C	**Decision-making about sexual relationships** For some people sexual intimacy is very important in a relationship, for others, it may not be as important. Can you tell me a little about your sexual relationship with your partner, how you deal with the possibility of HIV transmission, and how this has changed over the time you have been together?	

	1. How would you describe your sexual relationship?	- Are they sexually active with their partner, or anyone else
	2. How do you deal with the possibility of transmission within your relationship?	- What are their current sexual protection strategies, if any, and when/why are particular strategies preferred<br>- How were these decisions discussed and made, as a couple<br>- How do they think their partner feels about these practices<br>- Are they concerned about other sexually transmitted illnesses<br>- How would they say that their sexual practices are related to their overall relationship satisfaction
	3. How has this changed since you first met?	- Have things always been this way, or how is their current attitude/practice toward protection different as compared to an earlier time in the relationship<br>- How would they compare their sexual health/experiences in the current relationship to prior relationship(s)
	4. Tell me about HIV testing within your relationship.	- For HIV-negative partner, do they talk to their partner about HIV testing; is this something that they do on a regular basis; what motivates them to take the test
	5. What are your thoughts about the drugs called pre-exposure prophylaxis or PrEP? What about treatment as prevention or TasP?	- What do they know about these technologies<br>- How/have they discussed it with their partner<br>- What does their partner think/feel<br>- Is this something they have investigated, considered or employed<br>- If so, when, how often, and what motivated them to do so<br>- If not, would they consider using these strategies in the future; why/not<br>If they do not know what it is, describe it and probe whether it is something they could be beneficial in their relationship/would consider its use

D	Perceived needs of self/partner(s) and acceptability/uptake of health/social services/support<br>HIV prevention information, services and treatment have changed a great deal over the years. The next few questions are about your use of health and support services when you have questions or HIV-related health or support needs.	
	1. Tell me about who you turn to if you experience health issues, or need help or information about your health? For example, any health care or community services you access.	- do they use informal or formal networks (HIV-clinics or regular GP)<br>For **HIV-negative partner**, does their doc know they are in a serodiscordant relationship<br>-Do they talk about their HIV-risk management with their own Doc<br>-why/do they accompany their HIV+ve partner to their HIV-clinic visits<br>- how do they go have their questions HIV answered or to keep up-to-date on current health/HIV care<br>- What has been their experience with medical and social support - what about their use of community or social services<br>- Ask for examples – where do they go, and why (e.g., physical spaces, clinics, community organizations, ASOs, online websites/forums/blogs, peer groups, confidantes, relatives)<br>- How well do they think their needs are being met
	2. What about your relationship? Who would you turn to if you needed support or advice?	- have they experienced challenges to a relationship because of serodiscordance<br>do they use informal or formal networks<br>- what about their use of community or social services<br>- does being in a serodiscordant relationship lead them to turn to different resources than a seroconcordant one would<br>- Have they reached out to religious or faith-based communities<br>- How/do these services or communities meet their needs, what do they find helpful<br>- How/do they discuss their relationship at these venues<br>How well do they think their needs are being met<br>- How has this changed over time
	3. What sort of other social supports would you like to have available, if possible?	-are they satisfied with the assistance that they do have<br>-do they have any needs that they feel are unmet<br>-what would they like to see available<br>- -How has this changed over time

	4. What social supports do you suppose your partner would want or benefit from?	- How are their needs being met, emotionally and medically with regards to HIV or otherwise<br>- What is missing<br>- How/do they involve their partner<br>- What do they think are their partner's needs<br>- How do they think their partner's needs are/not being met
	5. Tell me about your family and friends, how many of them know you are in a serodiscordant relationship?	- How/do they discuss their relationship with others<br>- Have they disclosed the serodiscordant nature of their relationship<br>- Who do they disclose to (e.g., family, friends)<br>- What motivated them to disclose or not, how do they decide whether to disclose to someone else – is there an actual decision made about disclosure, are both partners involved in the decision<br>- How do their family or friends respond to or support one or both partners, or the relationship as a whole<br>- How has this changed over time
	6. Do either of you have any children?<br>- If yes, how do you talk about your relationship with them?<br>-If no, have you thought about having children?	- If yes, are the children from the current or prior relationship(s); to what extent are they involved in each others' lives; what have they shared about the relationship, and why; what was the response; how has this changed over time<br>- If no, how/have they discussed having children within their relationship; how do they think their partner thinks/feels; did they discuss this with anyone else (e.g., doctor, friend), and if so, what was the response; have they investigated, considered or employed any specific methods for conception; if they have used them – how satisfied were they with it, if not, difficulties did they encounter

E	Other contextual considerations (social networks, social norms, finances, policies/laws)<br>The next few questions are about your own community and broader society and the how they impact on your life and relationship	
	1. Tell me a little about the community in which you live. What do people in your community think and know about HIV?”	Note if asked, by community we mean the people that one interacts with on a daily basis, such as your friends, neighbours, and acquaintances<br>- how integrated are they into their community<br>- What general views do they hold about their community<br>- How is HIV perceived by the people they live/work with and by close confidantes/relatives
	2. Do you discuss your relationship with anyone in the community? What do you say?	- How do they speak about their relationship to others, and to whom (e.g., is it discussed in the context of HIV)<br>- If their/partner’s HIV status was disclosed to others, how did that happen, to whom, and what was the response<br>- If not disclosed, how do they think people would feel if they knew this was a serodiscordant relationship<br>- What types of things encourage them to disclose or avoid disclosure within their social networks<br>- How/do they know/socialize with other (serodiscordant/concordant) couples<br>- Have they ever moved or changed their involvement with the community because of the perception of HIV in their community
	3. How do you manage your financial needs? Are you currently working?	- If yes, ask about what they do, and what support they receive in terms of health benefits for self and partner<br>- If not, ask about prior work experiences just before and after HIV diagnosis; how/when/why did work end, and how did this affect this/prior relationship(s)<br>- How do they make ends meet<br>- What does their partner do<br>- How/has HIV restricted their ability to work/earn
	4. How do you think the law or government policies affect your relationship(s)? For example, have you heard about the criminalization of HIV nondisclosure? What are your thoughts about that?	- How/are Canadian laws/policies are broached/discussed within their relationship(s)<br>- What are their thoughts about particular policies such as the criminalization of HIV nondisclosure<br>- What does their partner think

<table>
<tr><td></td><td></td><td>- How/have particular laws affected disclosure decisions, intimacy or sexual practices within their relationship(s)
- Is their relationship affected by other laws or policies (e.g., in relation to their gender, sexual orientation, marital/common-law status)</td></tr>
<tr><td></td><td></td><td></td></tr>
<tr><td colspan="3">Wrap-up
We are now almost at the end of our interview. Thank you so much for sharing your thoughts. Is there anything you would like to talk about that we haven’t already talked about?

Please tell me what do you think makes your relationship work, in general? (end interview on an aspirational, positive note)

Would you like to add anything further? Thank you so much for your time.</td></tr>
<tr><td></td><td></td><td></td></tr>
</table>

Qualitative Interview Topic Guide – Current Relationships

HIV-**Positive** Partner

Introduction (After obtaining informed consent)<br>We are interested in learning about your thoughts and experiences while involved in your current relationship; and how your relationship has changed over the course of time. Remember, there are no right or wrong answers, we are interested in your personal thoughts and experiences. And thanks in advance for your time and participation.		
A	History and characterization of serodiscordant relationships<br>First we would like to know a bit about your current relationship, for example how and when your relationship started, and when and how HIV entered your relationship.	
	Questions	Probes
	5. Can you tell me a little about your current relationship?	- How do they describe their current relationship<br>- Are they living together/married; if not, how often do they meet, where do they meet, and what do they do<br>- What is the nature and history of their current relationship – how would they describe their partner; how/when did they meet; are they sexually and/or emotionally intimate; are they in a heterosexual, same sex, bisexual or transgendered relationship<br>-
	6. Are you seeing anyone else? Can you tell me about that relationship?	- Are they involved in any concurrent relationship(s)<br>- What is the nature and history of their other relationship(s) – how would they describe their other partner(s); how/when did they meet; are they sexually and/or emotionally intimate; are they in a heterosexual, same sex, bisexual or transgendered relationship<br>- Do they think their partner is involved in any concurrent relationships, how do they feel about that
	7. Can you tell me about your previous relationships?	- For HIV-positive partner, focus on relationships(s) during and post HIV diagnosis; how/did things change in relation to HIV diagnosis/disclosure<br>- How/why did the relationship(s) end<br>- How are things different in the current relationship
	8. Can you tell me about when you first faced HIV in the relationship?	- When in the relationship (or before) was the HIV-positive partner diagnosed<br>- When and how did the HIV-negative partner become aware of it<br>- How did that go Did it change over time<br>-

B	Satisfaction, challenges to wellbeing (individually and as a couple) and mechanisms of resiliency<br>Couples can experience many different challenges or issues in their relationships, including HIV, what types of challenges have you experienced in your relationship, and have you been able to overcome them?	
	5. Can you tell me about any issues or challenges that you have experienced in your current relationship?	- What were some of their early experiences in the relationship<br>- What about more recent relationship challenges/issues<br>- Focus on both HIV and non-HIV related issues<br>- What challenges does serodiscordance bring over and above that of gender, sexual orientation and HIV for a person not in a relationship?<br>- Ask for examples – what was the most recent issue that came up, what was the last argument about<br>- Suggest examples if none forthcoming (e.g., general relationship trials/conflicts, gender/sexual orientation issues, experience during HIV diagnosis/disclosure, treatment initiation/adherence/side effects, early/current sexual practices and protective strategies, concerns/fears about transmission, decisions about children/conception)<br>- What are their thoughts/feelings about these issues/challenges<br>- How/do they affect their mood/day-to-day life or sexual/relationship intimacy<br>- How do they think their partner perceives these issues/challenges
	6. How have you dealt with these issues?	- When/why did particular issues emerge, and how were they dealt with<br>- What issues were successfully resolved and how<br>- How were they managed or dealt with by the other partner<br>- Did they draw upon support from outside the relationship (e.g., friends, family, professional services)?<br>- Did similar issues/challenges arise in earlier serodiscordant relationships? If so how did they differ this time? Did they lead to break-ups in previous relationships?
	7. How do you think your current relationship is going?	- On what do they base their assessment? How can they tell when things are going well or not? What do they expect, need, want to be content/happy?<br>- Has HIV changed their conception of what they are looking for in a relationship?<br>- How has that satisfaction changed over time, how did it change with challenges related to HIV and non-HIV-related challenges<br>- How do they think their partner views their relationship now, and over the same issues and challenges<br>- If going well, why do they think it is going so well? If not going well, why do they think that is?

	8. Tell me a little about your/partner's current health. How do you think this has changed since your diagnosis?	- How is their physical health and general mood<br>- What specific issues/concerns do they have in relation to HIV and otherwise<br>- For HIV-positive partner, How do they manage treatment/adherence/medical appointments<br>-
C	**Decision-making about sexual relationships** For some people sexual intimacy is very important in a relationship, for others, it may not be as important. Can you tell me a little about your sexual relationship with your partner, how you deal with the possibility of HIV transmission, and how this has changed over the time you have been together?	
	6. How would you describe your sexual relationship?	- Are they sexually active with their partner, or anyone else
	7. How do you deal with the possibility of transmission within your relationship?	- What are their current sexual protection strategies, if any, and when/why are particular strategies preferred<br>- How were these decisions discussed and made, as a couple<br>- How do they think their partner feels about these practices<br>- Are they concerned about other sexually transmitted illnesses<br>- How would they say that their sexual practices are related to their overall relationship satisfaction
	8. How has this changed since you first met?	- Have things always been this way, or how is their current attitude/practice toward protection different as compared to an earlier time in the relationship<br>- How would they compare their sexual health/experiences in the current relationship to prior relationship(s)
	9. Tell me about HIV testing within your relationship.	- For **HIV-positive partner**, do they talk to their partner about HIV testing; is this something that the partner does on a regular basis; how does it make them feel<br>-
	10. What are your thoughts about the drugs called pre-exposure prophylaxis or PrEP? What about treatment as prevention or TasP?	- What do they know about these technologies<br>- How/have they discussed it with their partner<br>- What does their partner think/feel<br>- Is this something they have investigated, considered or employed<br>- If so, when, how often, and what motivated them to do so<br>- If not, would they consider using these strategies in the future; why/not<br>If they do not know what it is, describe it and probe whether it is something they could be beneficial in their relationship/would consider its use

D	Perceived needs of self/partner(s) and acceptability/uptake of health/social services/support<br>HIV prevention information, services and treatment have changed a great deal over the years. The next few questions are about your use of health and support services when you have questions or HIV-related health or support needs.	
	7. Tell me about who you turn to if you experience health issues, or need help or information about your health? For example, any health care or community services you access.	- do they use informal or formal networks (HIV-clinics or regular GP)<br>- how do they go have their questions HIV answered or to keep up-to-date on current health/HIV care<br>- What has been their experience with medical and social support - what about their use of community or social services<br>- Ask for examples – where do they go, and why (e.g., physical spaces, clinics, community organizations, ASOs, online websites/forums/blogs, peer groups, confidantes, relatives)<br>- How well do they think their needs are being met
	8. What about your relationship? Who would you turn to if you needed support or advice?	- have they experienced challenges to a relationship because of serodiscordance<br>do they use informal or formal networks<br>- what about their use of community or social services<br>- does being in a serodiscordant relationship lead them to turn to different resources than a seroconcordant one would<br>- Have they reached out to religious or faith-based communities<br>- How/do these services or communities meet their needs, what do they find helpful<br>- How/do they discuss their relationship at these venues<br>How well do they think their needs are being met<br>- How has this changed over time
	9. What sort of other social supports would you like to have available, if possible?	-are they satisfied with the assistance that they do have<br>-do they have any needs that they feel are unmet<br>-what would they like to see available<br>-How has this changed over time
	10. What social supports do you suppose your partner would want or benefit from?	- How are their needs being met, emotionally and medically with regards to HIV or otherwise<br>- What is missing<br>- How/do they involve their partner<br>- What do they think are their partner's needs<br>- How do they think their partner's needs are/not being met

	11. Tell me about your family and friends, how many of them know you are in a serodiscordant relationship?	- How/do they discuss their relationship with others<br>- Have they disclosed the serodiscordant nature of their relationship<br>- Who do they disclose to (e.g., family, friends)<br>- What motivated them to disclose or not, how do they decide whether to disclose to someone else – is there an actual decision made about disclosure, are both partners involved in the decision<br>- How do their family or friends respond to or support one or both partners, or the relationship as a whole<br>- How has this changed over time
	12. Do either of you have any children?<br>- If yes, how do you talk about your relationship with them?<br>-If no, have you thought about having children?	- If yes, are the children from the current or prior relationship(s); to what extent are they involved in each others' lives; what have they shared about the relationship, and why; what was the response; how has this changed over time<br>- If no, how/have they discussed having children within their relationship; how do they think their partner thinks/feels; did they discuss this with anyone else (e.g., doctor, friend), and if so, what was the response; have they investigated, considered or employed any specific methods for conception; if they have used them – how satisfied were they with it, if not, difficulties did they encounter

E	<u>Other contextual considerations (social networks, social norms, finances, policies/laws)</u><br>The next few questions are about your own community and broader society and the how they impact on your life and relationship	
	5. Tell me a little about the community in which you live. What do people in your community think and know about HIV?"	Note if asked, by community we mean the people that one interacts with on a daily basis, such as your friends, neighbours, and acquaintances<br>- how integrated are they into their community<br>- What general views do they hold about their community<br>- How is HIV perceived by the people they live/work with and by close confidantes/relatives

	6. Do you discuss your relationship with anyone in the community? What do you say?	- How do they speak about their relationship to others, and to whom (e.g., is it discussed in the context of HIV)<br>- If their/partner's HIV status was disclosed to others, how did that happen, to whom, and what was the response<br>- If not disclosed, how do they think people would feel if they knew this was a serodiscordant relationship<br>- What types of things encourage them to disclose or avoid disclosure within their social networks<br>- How/do they know/socialize with other (serodiscordant/concordant) couples<br>- Have they ever moved or changed their involvement with the community because of the perception of HIV in their community
	7. How do you manage your financial needs? Are you currently working?	- If yes, ask about what they do, and what support they receive in terms of health benefits for self and partner<br>- If not, ask about prior work experiences just before and after HIV diagnosis; how/when/why did work end, and how did this affect this/prior relationship(s)<br>- How do they make ends meet<br>- What does their partner do<br>- How/has HIV restricted their ability to work/earn
	8. How do you think the law or government policies affect your relationship(s)? For example, have you heard about the criminalization of HIV nondisclosure? What are your thoughts about that?	- How/are Canadian laws/policies are broached/discussed within their relationship(s)<br>- What are their thoughts about particular policies such as the criminalization of HIV nondisclosure<br>- What does their partner think<br>- How/have particular laws affected disclosure decisions, intimacy or sexual practices within their relationship(s)<br>- Is their relationship affected by other laws or policies (e.g., in relation to their gender, sexual orientation, marital/common-law status)

Wrap-up

We are now almost at the end of our interview. Thank you so much for sharing your thoughts. Is there anything you would like to talk about that we haven't already talked about?

Please tell me what do you think makes your relationship work, in general? (end interview on an aspirational, positive note)

Would you like to add anything further? Thank you so much for your time.

Qualitative Interview Topic Guide – PAST mixed-status Relationship

Person was HIV-Negative at the time of the relationsip

Introduction (After obtaining informed consent)<br>We are interested in learning about your thoughts and experiences while involved in your most-recent relationship with a person who was HIV-positive. We will ask questions about the relationship, how it changed over time, and how it ended. Remember, there are no right or wrong answers, we are interested in your personal thoughts and experiences. And, thanks in advance for your time and participation.<br><br>Since we'll be talking about a relationship in your past, and you may or may not have has current relationships, so let's start by working out how it is easiest for us to refer to your most recent sero-different partner. Some possibilities:<br>- Choose a pseudonym,<br>- refer to the person by the first initial of their first name,<br>- refer to the person as your 'past partner' or 'former partner', or 'positive partner'<br>- only use pronouns such as 'he,' 'she,' 'they'		
A	History and characterization of serodiscordant relationships<br>First we would like to know a bit about your relationship with ____, for example how and when your relationship started, when and how HIV entered your relationship, and how the relationship ended.	
	Questions	Probes
1.	Can you tell me a little about your relationship?	- How do they describe their past SD relationship<br>- were they living together/married; if not, how often did they meet, where did they meet, and what did they do together, and how often<br>- What was the nature and history of their relationship – how would they describe their partner; were they sexually and/or emotionally intimate; was it a heterosexual, same sex, bisexual or transgendered relationship

2.	Were they seeing anyone else at the same time? Can you tell me about that/those relationship(s)?	- Were they involved with other partners at the same time as this past relationship<br>- What was the nature and history of their other relationship(s) – how would they describe their other partner(s); how/when did they meet; are they sexually and/or emotionally intimate; are they in a heterosexual, same sex, bisexual or transgendered relationship.<br>- were any of them group sex<br>- Was their past partner aware that they were with others? (was it an open relationship) what did their partner think about them having other partners<br>- Do they think their past partner was involved in any concurrent relationships, how did they feel about that
3.	Can you tell me about when you first faced HIV in the relationship?	- For HIV negative partner: Were their partner positive when the relationship started or diagnosed during it<br>- if during the relationship, how did you react to their diagnosis<br>- When and how did you partner become aware of your partner's HIV (when and how disclosed, reaction to disclosure)<br>- How did that go, Did their reaction change over time<br>o If it changed, how did it change, how long did it take for them to change<br>- How much was HIV an issue for you by the time you broke up; how much of an issue was it for your partner<br>-
4.	How did the relationship come to an end?	- Who broke up with whom, or is the person deceased,<br>- how did the breakup occur, what were the major differences or problems<br>- When they broke up, was it expected, or did it come out of nowhere<br>- Did you feel that HIV was part of why you broke up? How?<br>-
5.	Can you tell me about your relationships before the one we are focusing on today?	- Had they been in a SD relationship before the one with the past partner,<br>- For HIV-negative partner: focus on nature of prior relationships in general, and any previous serodiscordant relationships, if they had them<br>- Did anything from pervious SD relationships help them in their relationship with their partner (HIV knowledge, disclosure, etc)<br>- How/why did the relationship(s) end, did they end for similar reasons to the "past partner"<br>- How did things differ in the "past" relationship<br>-

6.	What about relationships since you and your past partner broke-up, have you had any serious or casual relationships?	- Have they been in any serious or casual, SD or concordant<br>- Would they consider being in a SD relationship again; why, why not<br>- Has HIV changed their conception of what they are looking for in a relationship? How<br>- Would a person's use of HIV treatment or viral load have an impact on your partner /relationship choice<br>- Have you actively avoided SD relationships since your "past" relationship ended
B	Satisfaction, challenges to wellbeing (individually and as a couple) and mechanisms of resiliency<br>People can experience many different challenges or issues in their relationships, including HIV, what types of challenges did you experience in your relationship, and were you able to overcome them?	
1.	Would you say you were happy in your 'past" relationship?	- for the most part, were they mostly content? Looking back on it, and while they were in it.
2.	Can you tell me about any challenges or issues that you have experienced in your "past" SD relationship?	- What were some of their early experiences in the relationship<br>- What about more recent relationship challenges/issues<br>- Focus on both HIV and non-HIV related issues<br>- What challenges does serodiscordance bring over and above that of gender, sexual orientation and HIV for a person in a relationship?<br>- Ask for examples – what was the most recent issue that came up, what was the last argument about; what were their most common disagreements or arguments about<br>- Suggest examples if none forthcoming (HIV-related examples: gender/sexual orientation issues, experience during HIV diagnosis/disclosure, treatment initiation/adherence/side effects, sexual practices and protective strategies, concerns/fears about transmission, decisions about children/conception; acceptance of partner by family and friends)<br>- (non-HIV related examples: – general relationship trials/conflicts, other partners, general health, finances, substance use, household chores, children, etc)<br>- What are their thoughts/feelings about these issues/challenges<br>- How/do they affect their mood/day-to-day life or sexual/relationship intimacy<br>- How do they think their partner perceives these issues/challenges<br>- Try to find out if they feel that HIV was one of the major challenges in their relationship or was it one of many, or not really an issue at all

3.	How did you deal with these issues? (how did they solve them or get past them if not solved)	- When/why did particular issues emerge, and how were they dealt with<br>- What issues were successfully resolved and how<br>- What sorts of issues were they unable to get past<br>- How were they managed or dealt with by the other partner<br>- Did they draw upon support from outside the relationship (e.g., friends, family, professional services)? Was their difference in support if it was an HIV-related issue vs other types of issues
4.	If in a current relationship, (SD or otherwise) How does your current relationship differ from the "past" relationship in terms of disagreements and problems that arise?	- Have similar issues/challenges arisen your current relationship? If not, why do they think that is?<br>- what's different about the problems/ issues they face, do they experience the same issues, others<br>- If they have had a SD relationship since the 'past' relationship, is/was HIV an issue in the relationship? If different, describe how it is different<br>-
5.	How did your relationship satisfaction change over time?	- How did their relationship satisfaction change over time, how did it change with challenges related to HIV and non-HIV-related challenges<br>- How do they think their partner viewed their relationship at the time and over the same issues and challenges<br>-
6.	Tell me a little about your health, and your partner's health, at the time of the relationship?	- For HIV-negative partner: … your health at the time of the relationship, did it change during the relationship, how did you and your partner deal with health issues you faced<br>- How was your partner's health at the time of the relationship; were they on HIV medications? How was their viral load? How did that impact the relationship?<br>- How often and when/why do they go to their partner's medical appointments and or HIV-support sessions with the partner<br>- How did they manage treatment/adherence/medical appointments/cost?<br>- How did the partner's health impact on the negative partner<br>- How did HIV and other health issues affect the relationship (satisfaction/intimacy, etc)<br>- What specific concerns do they have in relation to HIV and other health concerns

C	Decision-making about sexual relationships<br>Now we will move on to talk about sex within your "past" relationship. For some people sexual intimacy is very important in a relationship, for others, it may not be as important. Can you tell me a little about your sexual relationship with your partner, how did you deal with the possibility of HIV transmission, and whether your sexual behaviour with your partner changed over the time you were together?	
1.	How would you describe your sexual relationship?	- Were they sexually active with their partner, or anyone else<br>- Were their sex drives similar, affected by HIV, did their sex drive change over time
2.	How did you deal with the possibility of transmission within your relationship?	- What were their sexual protection strategies, if any, when they first got together, and when/why are particular strategies preferred; did they both agree on condom use? Birth control (if heterosexual)?<br>- How were these decisions made, discussed, as a couple, did one partner have more influence in the decision<br>- Did their sexual behaviour/risk level change over time? How did that happen<br>- How do they think their partner felt about these practices<br>- Were they concerned about other sexually transmitted illnesses<br>- How would they say that their sexual practices and satisfaction were related to their overall relationship satisfaction
3.	How did this change during the relationship and after?	- Were things always been this way, or how is their current attitude/practice toward protection different when compared to the time of the relationship<br>- Was there any difference in safer sex practices if undetectable vs. detectable; did that have an impact on the relationship, sexual satisfaction<br>- How would they compare their sexual health/experiences in their current relationships to their "past" relationship
4.	Tell me about HIV testing within your relationship.	- For HIV-negative partner: did they talk to their partner about HIV testing;<br>- Is this (testing AND talking about it with partner) something that they did on a regular basis;<br>- What motivated them to take the test, how do they know it is time to take a test?<br>- Do they still test as often since the relationship? And for the same or other reasons

5.	What are your thoughts about the drugs called pre-exposure prophylaxis or PrEP?	- If they do not know what it is, describe it and probe whether it is something they think would have been beneficial in their "past" relationship and/or would have considered<br>- Was PrEP available at the time of their relationship? What did they know about it<br>- Did they discuss it with their partner<br>- What did their partner think/feel<br>- Is this something they investigated, considered, or employed<br>- If so, what motivated them to do so<br>- If not, would they consider using these strategies in current or future SD relationships, why/not<br>-

D	Perceived needs of self/partner(s) and acceptability/uptake of health/social services/support<br>HIV prevention information, services and treatment have changed a great deal over the years. The next few questions are about your use of health and support services when you have questions or HIV-related health or support needs.	
1.	Tell me about who you turn to if you experience health issues, or need help or information about your health? For example, any health care or community services you access.<br>(start out in the present)	- Do they use informal or formal networks (regular GP, online, friends/family, other)<br>- When they currently have HIV-related questions, how do they get them answered, or stay up-to-date on current health/HIV care<br>- What has been their experience with medical and social support - what about their use of community or social services<br>- Ask for examples – where do they go, and why (e.g., physical spaces, clinics, community organizations, s, online websites/forums/blogs, peer groups, confidantes, relatives)<br>- How well do they think their health needs are being met
2.	Did you have, use, or need these or any other supports at the time of your "past" relationship?	- Did they have other supports at the time of their relationship that they do not access now (e.g., Positive partner's physician, ASO, different on-line services)<br>- How did they make use of them at the time of the relationship?<br>- If they did not use, why not? Would they have liked to?<br>- For **HIV-negative partner**, did their doc/GP know they were in a SD relationship<br>-Did they talk about their HIV-risk management with their own Doc<br>- Why/did they accompany their HIV-positive partner to their HIV-clinic visits

3.	What about your relationship? Who did you turn to if you needed support or advice about your relationship?	- did they experience challenges to their relationship because of HIV or serodiscordance<br>- what informal or formal networks did they use (how /where did their partner get support and information)<br>- what about their use of community or social services<br>- did being in a SD relationship lead them to turn to different resources than a seroconcordant one would<br>- Have they reached out to religious or faith-based communities<br>- How/did these services or communities meet their needs, what do they find helpful<br>- How/did they discuss their relationship at these venues<br>- How well do they think their relationship needs were being met<br>- How has this changed over time,
4.	What sort of other social supports would you have liked to have had available to YOU at the time of your "past" relationship, in an ideal world?	- Were they satisfied with the assistance that they did have<br>- What needs did they have that they felt were unmet (e.g., emotional, medical, and regarding HIV information)<br>- What services might have helped with any needs they had, that were unmet / What would they like to see available<br>- Can they think of anything that would have helped your partner adjust more to being in a relationship with someone who is HIV-positive<br>- Did these service needs change over time in your relationship or with later relationships
5.	-	-
6.	Tell me about your family and friends, how many of them knew you are in a serodiscordant relationship?	- How/did they discuss their relationship with others<br>- Did they disclose the serodiscordant nature of their relationship (that their partner was HIV-positive)<br>- Who did they disclose to (e.g., family, friends) at the time of the relationship<br>- What motivated them to disclose or not, how did they decide whether to disclose to someone else – was there an actual decision made about disclosure, were both partners involved in the decision<br>- How did their family or friends respond to or support one or both partners, or the relationship as a whole<br>- How did this changed over time

E	Other contextual considerations (social networks, social norms, finances, policies/laws)<br>The next few questions are about your own community and broader society and the how they impact on your life and relationship	
1.	Tell me a little about the community in which you currently live. What do people in your community think and know about HIV?"<br><br>(ask about current situation)	Note if asked, by community we mean the people that one interacts with on a daily basis, such as your friends, neighbours, and acquaintances<br>- how integrated are they into their community<br>- What general views do they hold about their community<br>- How is HIV perceived by the people they live/work with and by close confidantes/relatives<br>- Is this the same community that they were part of during their "past" relationship –If no, how did it differ
2.	Did you discuss your "past" SD relationship with anyone in the community? What would you say?	- If their partner's HIV status was disclosed to others, how did that happen, to whom, and what was the response<br>- How did they speak about their relationship to others, and to whom (e.g., was it discussed in the context of HIV)<br>- Did they feel the need to actively hide their partner's HIV status, why, how did that make them feel<br>- If not disclosed, how do they think people would feel if they knew this was a SD relationship (was HIV stigma a concern for them or their "past" partner)<br>- How/did they know/socialize with other (SD/concordant) couples<br>- Have they ever moved or changed their involvement with the community because of the perception of HIV in their community; Did this happen when you were with your "past" partner<br>- What types of things encouraged them to disclose or avoid disclosure within their social networks
3.	How was your financial situation when you were with your "past" partner?	- Were you working, receive benefits from own work or partners?<br>- What did their partner do (work/disability, etc)<br>- Were the costs of their partner's HIV meds covered, how<br>- Were finances tight; If yes, how did they make ends meet<br>- How/did HIV restrict their partner's ability to work/earn<br>- How did financial situation affect their relationship

4.	How do you think the law or government policies affected your relationship(s)? For example, have you heard about the criminalization of HIV nondisclosure? What are your thoughts about that?	- How/are Canadian laws/policies are broached/discussed within their relationship(s)<br>- What are their thoughts about particular policies such as the criminalization of HIV nondisclosure<br>- What does their partner think<br>- How/have particular laws affected disclosure decisions, intimacy or sexual practices within their relationship(s); impact of these impacts upon relationship success/satisfaction<br>- Was their "past" relationship (or any since) affected by other laws or policies (e.g., in relation to their gender, sexual orientation, marital/common-law status)
	Wrap up	
1.	That's the end of the questions that I have for you. Before we move on to the gift card, is there anything else you would like to talk about that we have not covered yet?	
Thanks so much for sharing your time and your thoughts. I'll turn off the tape recorder now and we can arrange for your gift card.		

Qualitative Interview Topic Guide – PAST mixed-status Relationship

Person was HIV-Positive at the time of the relationsip

Introduction (After obtaining informed consent)

We are interested in learning about your thoughts and experiences while involved in your most-recent relationship with a person who had a different HIV-status than you. We will ask questions about the relationship, how changed it over time, and how it ended. Remember, there are no right or wrong answers, we are interested in your personal thoughts and experiences. And, thanks in advance for your time and participation.

Since we'll be talking about a relationship in your past, and you may or may not have has current relationships, so let's start by working out how it is easiest for us to refer to your most recent sero-different partner. Some possibilities:

- Choose a pseudonym,
- refer to the person by the first initial of their first name,
- refer to the person as your 'past partner' or 'former partner', or 'negative partner'
- only use pronouns such as 'he,' 'she,' 'they'

A	History and characterization of serodiscordant relationships<br>First we would like to know a bit about your relationship with ____, for example how and when your relationship started, when and how HIV entered your relationship, and how the relationship ended.	
	Questions	Probes
1.	Can you tell me a little about your relationship?	- How do they describe their past SD relationship<br>- were they living together/married; if not, how often did they meet, where did they meet, and what did they do together<br>- What was the nature and history of their relationship – how would they describe their partner; were they sexually and/or emotionally intimate; was it a heterosexual, same sex, bisexual or transgendered relationship
2.	Were they seeing anyone else at the same time? Can you tell me about that/those relationship(s)?	- Were they involved with other partners at the same time as this past relationship<br>- What was the nature and history of their other relationship(s) – how would they describe their other partner(s); how/when did they meet; are they sexually and/or emotionally intimate; are they in a heterosexual, same sex, bisexual or transgendered relationship.<br>- were any of them group sex<br>- Was their past partner aware that they were with others? (was it an open relationship) what did their partner think about them having other partners<br>- Do they think their past partner was involved in any concurrent relationships, how did they feel about that

3.	Can you tell me about when you first faced HIV in the relationship?	- For HIV positive partner: Were they positive when the relationship started or diagnosed during it<br>- if during the relationship, how did you react to your diagnosis<br>- When and how did your partner become aware of it (when and how disclosed, reaction to disclosure)<br>- How did that go, Did their reaction change over time<br>o If it changed, how did it change, how long did it take for them to change<br>- How much was HIV an issue for you by the time you broke up; how much of an issue was it for your partner<br>-
4.	How did the relationship come to an end?	- Who broke up with whom, or is the person deceased,<br>- how did the breakup occur, what were the major differences or problems<br>- When they broke up, was it expected, or did it come out of nowhere<br>- Did you feel that HIV was part of why you broke up? How?
5.	Can you tell me about your relationships before the one we are focusing on today?	- Had they been in a SD relationship before the one with the past partner,<br>- For HIV-positive partner, focus on relationships(s) during and post HIV diagnosis; how/did things change in relation to HIV diagnosis/disclosure<br>- Did anything from pervious SD relationships help them in their relationship with their partner (HIV knowledge, disclosure, etc)<br>- How/why did the relationship(s) end, did they end for similar reasons to the “past partner”<br>- How did things differ in the “past” relationship<br>-
6.	What about relationships since you and your past partner broke-up, have you had any serious or casual relationships?	- Have they been in any serious or casual, SD or concordant<br>- Would they consider being in a SD relationship again; why, why not<br>- Has HIV changed their conception of what they are looking for in a relationship? How<br>- Do you think that your HIV treatment or viral load have an impact on your relationships or partner choice
B	Satisfaction, challenges to wellbeing (individually and as a couple) and mechanisms of resiliency<br>People can experience many different challenges or issues in their relationships, including HIV, what types of challenges did you experience in your relationship, and were you able to overcome them?	

1.	Would you say you were happy in your 'past" relationship?	-for the most part, were they mostly content? Looking back on it, and while they were in it.
2.	Can you tell me about any challenges or issues that you have experienced in your "past" SD relationship?	- What were some of their early experiences in the relationship<br>- What about more recent relationship challenges/issues<br>- Focus on both HIV and non-HIV related issues<br>- What challenges does serodiscordance bring over and above that of gender, sexual orientation and HIV for a person in a relationship?<br>- Ask for examples – what was the most recent issue that came up, what was the last argument about; what were their most common disagreements or arguments about<br>- Suggest examples if none forthcoming (HIV-related examples: gender/sexual orientation issues, experience during HIV diagnosis/disclosure, treatment initiation/adherence/side effects, sexual practices and protective strategies, concerns/fears about transmission, decisions about children/conception, acceptance by partner's family and friends)<br>- (non-HIV related examples: – general relationship trials/conflicts, other partners, general health, finances, substance use, household chores. Children, etc)<br>- What are their thoughts/feelings about these issues/challenges<br>- How/do they affect their mood/day-to-day life or sexual/relationship intimacy<br>- How do they think their partner perceives these issues/challenges<br>- Try to find out if they feel that HIV was one of the major challenges in their relationship or was it one of many, or not really an issue at all
3.	How did you deal with these issues? (how did they solve them or get past them if not solved)	- When/why did particular issues emerge, and how were they dealt with<br>- What issues were successfully resolved and how<br>- What sorts of issues were they unable to get past<br>- How were they managed or dealt with by the other partner<br>- Did they draw upon support from outside the relationship (e.g., friends, family, professional services)? Was their difference in support if it was an HIV-related issue vs other types of issues

4.	If in a current relationship, (SD or otherwise) How does your current relationship differ from the "past" relationship in terms of disagreements and problems that arise?	- Have similar issues/challenges arisen your current relationship? If not, why do they think that is?<br>- what's different about the problems/ issues they face, do they experience the same issues, others<br>- If they have had a SD relationship since the 'past' relationship, is/was HIV an issue in the relationship? If different, describe how it is different<br>-
5.	How did your relationship satisfaction change over time?	- How did their relationship satisfaction change over time, how did it change with challenges related to HIV and non-HIV-related challenges<br>- How do they think their partner viewed their relationship at the time and over the same issues and challenges<br>-
6.	Tell me a little about your health, and your partner's health, at the time of the relationship?	- For HIV-positive partner: … your health at the time of the relationship, did it change during the relationship, how did you and your partner deal with health issues you faced, did they accompany you to appointments, support about HIV, etc?<br>- For HIV-positive partner: How do they manage treatment/adherence/medical appointments/cost<br>- How about your partner, how is their physical health and general mood<br>- any other health issues other than HIV, how did these other health issues affect the relationship (satisfaction/intimacy, etc)<br>- What specific concerns do they have in relation to HIV and other health concerns
C	Decision-making about sexual relationships<br>Now we will move on to talk about sex within your "past" relationship. For some people sexual intimacy is very important in a relationship, for others, it may not be as important. Can you tell me a little about your sexual relationship with your partner, how did you deal with the possibility of HIV transmission, and whether your sexual behaviour with your partner changed over the time you were together?	
1.	How would you describe your sexual relationship?	- Were they sexually active with their partner, or anyone else<br>- Were their sex drives similar, affected by HIV, did their sex drive change over time

2.	How did you deal with the possibility of transmission within your relationship?	- What were their sexual protection strategies, if any, when they first got together, and when/why are particular strategies preferred; did they both agree on condom use? Birth control (if heterosexual)?<br>- How were these decisions made, discussed, as a couple, did one partner have more influence in the decision<br>- Did their sexual behaviour/risk level change over time? How did that happen<br>- How do they think their partner felt about these practices<br>- Were they concerned about other sexually transmitted illnesses<br>- How would they say that their sexual practices and satisfaction were related to their overall relationship satisfaction
3.	How did this change during the relationship and after?	- Were things always been this way, or how is their current attitude/practice toward protection different when compared to the time of the relationship<br>- Was there any difference in safer sex practices if undetectable vs. detectable; did that have an impact on the relationship, sexual satisfaction<br>- How would they compare their sexual health/experiences in their current relationships to their “past” relationship
4.	Tell me about HIV testing within your relationship.	- For **HIV-positive partner**: did they talk to their partner about HIV testing; is this something that the partner did on a regular basis; how did they feel about their partner’s testing frequency, and the possibility they could become HIV-positive<br>-
5.	What are your thoughts about the drugs called pre-exposure prophylaxis or PrEP?	- If they do not know what it is, describe it and probe whether it is something they think would have been beneficial in their “past” relationship and/or would have considered<br>- Was PrEP available at the time of their relationship? What did they know about it<br>- Did they discuss it with their partner<br>- What did their partner think/feel<br>- Is this something they investigated, considered, or employed<br>- If so, what motivated them to do so<br>- If not, would they consider using these strategies in current or future SD relationships, why/not<br>-

D	Perceived needs of self/partner(s) and acceptability/uptake of health/social services/support<br>HIV prevention information, services and treatment have changed a great deal over the years. The next few questions are about your use of health and support services when you have questions or HIV-related health or support needs.	
1.	Tell me about who you turn to if you experience health issues, or need help or information about your health? For example, any health care or community services you access.<br>(start out in the present)	- Do they use informal or formal networks (HIV-clinics, regular GP, ASOs, friends/family)<br>- If they have HIV-related questions, how do they get them answered, or stay up-to-date on current health/HIV care<br>- What has been their experience with medical and social support - what about their use of community or social services<br>- Ask for examples – where do they go, and why (e.g., physical spaces, clinics, community organizations, ASOs, online websites/forums/blogs, peer groups, confidantes, relatives)<br>- How well do they think their needs are being met
2.	Did you have these supports at the time of your "past" relationship?	- How did they make use of them at the time of the relationship?<br>- If they did not use, why not? Would they have liked to?<br>- Did they have other supports at the time of their relationship that they do not access now
3.	What about your relationship? Who did you turn to if you needed support or advice about your relationship?	- did they experience challenges to their relationship because of HIV or serodiscordance<br>- what informal or formal networks did they use<br>- what about their use of community or social services<br>- did being in a SD relationship lead them to turn to different resources than a seroconcordant one would<br>- Have they reached out to religious or faith-based communities<br>- How/did these services or communities meet their needs, what do they find helpful<br>- How/did they discuss their relationship at these venues<br>- How well do they think their relationship needs were being met<br>- How has this changed over time,

4.	What sort of other social supports would you have liked to have had available to YOU at the time of your "past" relationship, in an ideal world?	- Were they satisfied with the assistance that they did have<br>- What needs did they have that they felt were unmet<br>- What services might have helped with any needs they had that were unmet / What would they like to see available<br>- Did these service needs change over time in your relationship or with later relationships
5.	What social supports do you suppose your HIV-negative partner would have wanted or benefited from?	- How well were their needs met emotionally and medically with regard to HIV<br>- What do they think their partner need help with<br>- How were their needs met, emotionally, medically, and regarding HIV information (how /where did their partner get support and information)<br>- What was missing for them<br>- Can you think of anything that would have helped your partner adjust more to being in a relationship with someone who is HIV-positive
6.	Tell me about your family and friends, how many of them knew you are in a serodiscordant relationship?	- How/did they discuss their relationship with others<br>- Did they disclose the serodiscordant nature of their relationship<br>- Who did they disclose to (e.g., family, friends) at the time of the relationship<br>- What motivated them to disclose or not, how did they decide whether to disclose to someone else – was there an actual decision made about disclosure, were both partners involved in the decision<br>- How did their family or friends respond to or support one or both partners, or the relationship as a whole<br>- How did this changed over time

E	Other contextual considerations (social networks, social norms, finances, policies/laws)<br>The next few questions are about your own community and broader society and the how they impact on your life and relationship	
1.	Tell me a little about the community in which you currently live. What do people in your community think and know about HIV?"<br><br>(ask about current situation)	Note if asked, by community we mean the people that one interacts with on a daily basis, such as your friends, neighbours, and acquaintances<br>- how integrated are they into their community<br>- What general views do they hold about their community<br>- How is HIV perceived by the people they live/work with and by close confidantes/relatives<br>- Is this the same community that you were a part of during their "past" relationship –If no, how did it differ
2.	Did you discuss your "past" SD relationship with anyone in the community? What would you say?	- If their HIV status was disclosed to others, how did that happen, to whom, and what was the response<br>- How did they speak about their relationship to others, and to whom (e.g., was it discussed in the context of HIV)<br>- Did they feel the need to actively hide their HIV status, why, how did that make them feel<br>- If not disclosed, how do they think people would feel if they knew this was a SD relationship (was HIV stigma a concern for them or their "past" partner)<br>- How/did they know/socialize with other (SD/concordant) couples<br>- Have they ever moved or changed their involvement with the community because of the perception of HIV in their community; Did this happen when you were with your "past" partner<br>- What types of things encourage them to disclose or avoid disclosure within their social networks
3.	How was your financial situation when you were with your "past" partner?	- Were you working, receive benefits from own work or partners? Were the costs of your HIV meds covered?<br>- If not, ask about prior work experiences just before and after HIV diagnosis; how/when/why did work end,<br>- How/did HIV restrict their ability to work/earn<br>- Were finances tight; If yes, how did they make ends meet<br>- What did their partner do<br>- How did financial situation affect their relationship

4.	How do you think the law or government policies affected your relationship(s)? For example, have you heard about the criminalization of HIV nondisclosure? What are your thoughts about that?	- How/are Canadian laws/policies are broached/discussed within their relationship(s)<br>- What are their thoughts about particular policies such as the criminalization of HIV nondisclosure<br>- What does their partner think<br>- How/have particular laws affected disclosure decisions, intimacy or sexual practices within their relationship(s); impact of these impacts upon relationship success/satisfaction<br>- Was their “past” relationship (or any since) affected by other laws or policies (e.g., in relation to their gender, sexual orientation, marital/common-law status)
	Wrap up	
1.	That’s the end of the questions that I have for you. Before we move on to the gift card, is there anything else you would like to talk about that we have not covered yet?	
Thanks so much for sharing your time and your thoughts. I’ll turn off the tape recorder now and we can arrange for your gift card.		
