# Supplementary material for: “Live a normal life”: Constructions of resilience among people in mixed HIV status relationships in Canada
Source: PLoS One. 2023 Mar 8;18(3):e0281301. doi: 10.1371/journal.pone.0281301 (PMC9994671; doi:10.1371/journal.pone.0281301)
Supplement: S1 File — (PDF) [file pone.0281301.s002.pdf]

Data availability and publication consent

Re: PONE-D-22-15412
"Live a normal life": Constructions of resilience among people in mixed HIV status relationships in Canada

Competing interests:

Amrita Daftary is a section editor for PLOS Global Public Health and academic editor for PLOS One. Bertrand Lebouché has received grants for investigator-initiated studies from ViiV Healthcare, Merck, and Gilead; consulting fees from ViiV Healthcare, Merck, and Gilead. All other authors have no competing interests.

Consent for publication: All study participants provided informed consent prior to enrollment providing permission to publish anonymous quotes from their interviews.

Availability of data and material: De-identified data excerpts are presented within the paper, as a minimal dataset. There are ethical restrictions on sharing the complete de-identified study transcripts, due to the ability of one person to identify their partner's statements. The study consent letter included the statement: "Although we may use quotes from your interview in future publications or presentations, all quotes will be anonymous. Also, each quote will be general enough in nature that you will not be identifiable, even by your partner." Providing fully-transcribed interviews would allow one partner to easily identify their partner, and possibly other people. As a result, the data can only be made available to other researchers if terms of anonymity are met via data transfer agreements with the corresponding author.

Code availability: A full codebook is not available, as it is connected to other analyses published elsewhere.
