# Supplementary material for: “Live a normal life”: Constructions of resilience among people in mixed HIV status relationships in Canada
Source: PLoS One. 2023 Mar 8;18(3):e0281301. doi: 10.1371/journal.pone.0281301 (PMC9994671; doi:10.1371/journal.pone.0281301)
Supplement: Attachment — Submitted filename: PONE-D-22-15412.docx [file pone.0281301.s003.docx]

**Manuscript Number:** PONE-D-22-04872
**Manuscript Title:** The dermatology clinic may be an effective human immunodeficiency virus (HIV) screening service delivery point for provider-initiated testing and counseling (PITC) services in Malawi

**Summary**

The authors describe resilience among HIV mixed status couples in Canada. Findings presented in this manuscript comprise a sub-analysis of qualitative data drawn from Positive Plus One Mixed methods study. Qualitative results contained in this manuscript are from an original research and have not been published elsewhere. There are however, a few areas that the authors should work on to further improve the manuscript. These areas have been described below:

**Minor revisions**

**Abstract**

Page 3: The abstract has information on methodology and findings. Authors should include a statement on key conclusion from this research. They should also provide information on how they analysed qualitative data.

**Introduction**

Page 4: Second paragraph: The authors describes that ‘For mixed HIV-status couples, resilience is found to be closely bound to HIV treatment and prevention.” This statement ignore the fact that ‘resilience’ within the context of mixed status couples is a complex process comprising other important elements that shapes its. I suggest that the authors should provide a description of other factors that influence resilience in mixed status couples in addition to HIV treatment and prevention. Examples of these may include level of trust, the differences in economic position and power between couples, cultural context among others.

Page 5: Second paragraph: A statement that begins with ‘Recent work from Australia suggests that couples… ‘ should read ‘Recent work from Australia suggests that HIV mixed status couples …. “

**Methods**

The methods section does not provide information on how study participants were approached i.e. face-to-face, telephone, email. There is also no statement that describes the number of individuals that refused to participate in the study and the reasons for this.

The authors have also not provided a theoretical framework that informed their methodology.

The authors have not provided the credentials of individuals that collected qualitative data - i.e. gender, sexual orientation, qualifications - and whether or not their attributes mirrors those of the study participants.

The authors should provide information on the setting where qualitative data was collected - home or clinic or a neutral venue.

Furthermore, there is need for authors to describe whether sexual partners where present during interviews.

Authors do not describe how they ensured that they collected sufficient data for this analysis.

Page 6: Second paragraph: Authors write - ‘Transcripts were coded by two research team members (MY, MR) who led the thematic analysis, used an inductive approach, identifying patterns within and across data to generate emerging themes.’ It is useful to indicate whether or not MY and MR were coding the data together or they performed parallel data coding followed by a coding consolidation meeting. The authors should also describe if data coding was done manually or with the help of a computer soft ware.

**Results**

It will be quite interesting of the analysis presented in the results section compares the experiences of resilience between sexual partiners that are living in mixed status heterosexual relationships to those living in mixed status GBSMS relationships. If the experiences were the same, it will be good that this should be mentioned.

Pages 7&8; Paragraph 1 and 2: Most of the qualitative studies include the demographic characteristics in the methods section and not in results section since participants selection is usually done using non-probabilistic sampling techniques. Author should provide an explanation on why they have included these demographic characteristics within the results section.

Page 8; Paragraph 3: Authors states ‘timing of HIV in the relationship.’ This phrase has been presented in several sections of the manuscript. This phrase need to be clarified. Do they refer to the ‘timing of entry of HIV within the relationship’ or ‘the timing of learning that they are living in a mixed status relationship.’

Page 8; The theme titles ‘ feeling and being ‘normal’; entering intimate relationships, accessing information and services etc can be creatively presented. For example, access to reliable medical information and healthcare services; status disclosure, HIV stigma and acceptance etc. I think that themes presented in Figure 1 can been better sub-titles for themes of this manuscript.

***Sub-title : Assessing information***

Page 13. Paragraph 1: Replace the word ‘discordance’ with ‘mixed status’ or sero different’

Page 14. Authors states ‘Financial barriers, for example, were only partly alleviated by the public health system that offered basic HIV treatment services at no charge but limited access to allied healthcare services.” It is useful to mention a few of these ‘allied health care services’.

Quotes in the results section: It is useful to provide a much richer description of the characteristics of the individuals who provided these quotes. Eg. HIV positive heterosexual man, 30 years, employed, XX province.

**Discussion**

Page 23: Authors write “We uncovered how pharmaceutical technologies,…” In the result section, there is no mention of pharmaceutical technologies. It will therefore be important to indicate the names of these technologies which have also been presented in the results section.
